# Prevalence and risk factors of psychiatric disorders in early adolescence: 2004 Pelotas (Brazil) birth cohort

**DOI:** 10.1007/s00127-018-1516-z

**Published:** 2018-04-13

**Authors:** Carolina La Maison, Tiago N. Munhoz, Iná S. Santos, Luciana Anselmi, Fernando C. Barros, Alicia Matijasevich

**Affiliations:** 1 Department of Preventive Medicine, Faculty of Medicine FMUSP, University of São Paulo, São Paulo, Brazil; 2 Department of Psychology, Federal University of Pelotas, Pelotas, Brazil; 3 Postgraduate Program in Epidemiology, Federal University of Pelotas, Pelotas, Brazil; 4 Postgraduate Program in Health and Behavior, Catholic University of Pelotas, Pelotas, Brazil; 5 Centro de Pesquisas Epidemiológicas, Universidade Federal de Pelotas, Rua Marechal Deodoro, 1160, Caixa Postal 464, Pelotas, RS CEP: 96020-220 Brazil

**Keywords:** Mental disorders, Neurodevelopmental disorders, Prospective studies, Adolescent

## Abstract

**Purpose:**

The present study aimed to evaluate the prevalence of psychiatric disorders in early adolescence, to examine the distribution of psychiatric disorders by maternal and child characteristics and to evaluate the occurrence of psychiatric comorbidities.

**Methods:**

This was a prospective cohort study of all live births in the city of Pelotas, Brazil, in 2004 (*n* = 4231). A total of 3562 subjects were evaluated at 11 years of age. Psychiatric disorders were assessed using the Development and Well-Being Assessment. Crude and adjusted logistic regression was used to investigate risk factors for any psychiatric disorder.

**Results:**

According to DSM-5 criteria, the overall prevalence of psychiatric disorders was 13.2% (*n* = 471), 15.6% among the boys and 10.7% among the girls. The most common disorders were anxiety disorders (4.3%), any attention deficit/hyperactivity disorder (4.0%) and any conduct/oppositional disorder (2.8%). Low maternal education, smoking during pregnancy, the presence of moods symptoms during pregnancy or maternal chronic and severe depressive symptoms in the first years of the adolescent´s life, male gender, 5-min Apgar score < 7 at birth and preterm birth were associated with higher odds of any psychiatric disorder at age 11. Psychiatric comorbidities were observed in 107 subjects (22.7%), of whom 73, 24, and 10 had two, three, and four psychiatric diagnoses, respectively.

**Conclusions:**

Our results underscore the importance of psychiatric disorders as a prevalent condition in early adolescence, which has a direct impact on the planning of public policies and specific mental health care services in this age group.

**Electronic supplementary material:**

The online version of this article (10.1007/s00127-018-1516-z) contains supplementary material, which is available to authorized users.

## Introduction

Psychiatric disorders account for a significant proportion of the global burden of disease [1, 2]. Psychiatric disorders represent 9.8% of the burden of disease in low- and middle-income countries [3]. According to Kieling et al. [1], at least 10% of children and adolescents worldwide have a psychiatric disorder, a major cause of morbidity and mortality. Among individuals between 10 and 24 years of age, psychiatric disorders account for 45% of all years lived with disability [4]. In addition, psychiatric disorders and substance use have been found to be the main causes of disability-adjusted life-years (DALYs) among young people in high-income countries [5].

In adolescents, psychiatric disorders not only cause personal and family suffering, but also are recognized risk factors for substance abuse and criminality, as well as predicting negative outcomes in adulthood [6]. A significant proportion of psychiatric disorders diagnosed in adults have their roots in childhood and adolescence [7].

A recent systematic review and meta-analysis of the prevalence of psychiatric disorders in adolescents in 27 countries reported an overall prevalence of psychiatric disorders of 13.4% (95% CI 11.3–15.9). Having duly adjusted for methodological factors, the authors observed a great similarity in the frequency of psychiatric disorders among different countries and cultures [8]. Studies conducted in Brazil have reported the prevalence of psychiatric disorders in adolescence to range from 7 to 20%, depending on the region investigated, the level of exposure to risk factors, and the methodology used [1].

A wide variety of scales and questionnaires are available for the measurement of psychiatric disorders in low- and middle-income countries [9, 10]. Prioritizing either the sensitivity (screening criteria) or specificity (diagnostic criteria) of such tests can result in differences in measures of disease occurrence. Higher rates of psychiatric disorders are observed in studies that apply screening criteria. A systematic review and meta-analysis of the prevalence of major depressive disorder, including 116 studies, found that studies using symptom scales reported a higher prevalence of the disorder than did those using established criteria such as those outlined in the DSM-IV and ICD-10 [11].

There is little evidence of specific associations between particular risk factors and particular psychiatric disorders. The most reasonable explanation for the development of psychiatric disorders might be the combination, sequence, interaction of individual, familial, genetic, and environmental risk factors [12, 13]. Risk factors for psychiatric disorders range from the biological, genetic, and perinatal to the gender of the child, parental attachment, and trauma, as well as demographic and socioeconomic factors. Among the environmental risk factors associated with psychiatric disorders, the findings related to socioeconomic position are the most robust in epidemiological studies, which have shown an inverse association, a lower socioeconomic position increasing the probability of the individual developing a psychiatric disorder [14, 15].

The frequency and type of psychiatric disorder occurring during adolescence varies according to the gender of the individual. Various studies have suggested that, among adolescents, conduct/oppositional disorders and attention deficit/hyperactivity disorder (ADHD) are more common in males, whereas anxiety and depressive disorders are more common in females [16]. Externalizing problems—conduct disorder and oppositional defiant disorder (ODD)—tend to improve with age, whereas internalizing problems—anxiety and depression—tend to worsen [17].

Psychiatric disorders rarely occur in isolation, and comorbidity with other psychiatric disorders is common [18]. The presence of psychiatric comorbidities complicates the diagnostic process, having a significant impact on the natural history, prognosis, and treatment of the primary psychiatric disorder [19]. Two or more comorbid psychiatric disorders reportedly occur in approximately 16% of adolescents [20].

The present study had the following objectives: to evaluate the prevalence of psychiatric disorders in individuals at the beginning of adolescence (11 years of age); to examine the distribution of psychiatric disorders by gender, socioeconomic position (SEP) and other maternal and child characteristics; and to evaluate the occurrence of psychiatric comorbidities.

## Methods

### Setting and study design

The city of Pelotas is in the south of Brazil, within the state of Rio Grande do Sul. It has a population of approximately 328,000 inhabitants, of which 93.3% live in the urban area of the city. In 2010, the city had an adult literacy rate of 95.7%, higher than the 72.0% reported for Brazil as a whole, and the mean Human Development Index was 0.816, compared with 0.699 for the country as a whole.

In 2004, all children born at hospitals to mothers living within the urban area of the city of Pelotas were identified and their mothers were invited to participate in a cohort study. The follow-up group comprised 4231 children born to the mothers who agreed to participate in the study (refusals < 1%). A detailed description of the methodology employed can be found elsewhere [21]. Members of the Pelotas 2004 Birth Cohort were evaluated at various time points. The sixth follow-up evaluation of the cohort was conducted between May and October 2015 (follow-up rate of 87%), when the mean age of the subjects was 11.0 years (SD = 0.3).

### Evaluation of psychiatric disorders

The Development and Well-Being Assessment (DAWBA) combines closed and open-ended (qualitative descriptions) questions based on DSM-IV, DSM-5 and ICD-10 diagnoses [22]. The following diagnoses were assessed: separation anxiety disorder; specific phobia; social phobia; generalized anxiety disorder; post-traumatic stress disorder; panic disorder and agoraphobia; obsessive–compulsive disorder; depressive disorder, bipolar disorder, and ADHD; ODD; conduct disorder; autism spectrum disorders, eating disorders, and tic disorder, including three new DSM-5 diagnoses—body dysmorphic disorder, disruptive mood dysregulation disorder (DMDD), and binge-eating disorder. We employed the Portuguese-language version of the DAWBA, which has been cross-culturally adapted and validated for use in Brazil [23] which was administered to mothers or caregivers by trained psychologists. The training included a theoretical and a practical phase. The theoretical phase consisted of lessons on the various psychopathologies addressed in the DAWBA, the way the DAWBA instruments work, and the precautions to be taken in their application. The practical phase consisted of supervised interviews with the mothers of children treated at the Pediatric Outpatient Clinic or Psychiatric Clinic of the School of Medicine of the Federal University of Pelotas. The training lasted for 40 h. During the fieldwork, the psychologists were supervised on a weekly basis, their questions being answered and explanations about the DAWBA being repeated, the supervisory sessions, therefore, functioning as ongoing reinforcement of the training. The clinical evaluation of the total sample was performed by a psychologist, and a second independent psychologist evaluated 10% of the study sample. Both were trained in how to apply the DAWBA, in a standardized manner, by the child psychiatrist who had translated and validated the questionnaire for use in Brazil [24]. The inter-rater agreement was 91.2% for the presence of any psychiatric disorder, 75.9% for any anxiety disorder, 73.5% for any depressive disorder, 72.7% for ADHD, 72.9% for conduct disorder, 85.6% for any autism spectrum disorder, 59.5% for any eating disorder, and 52.4% for any tic disorder. We described the DAWBA diagnoses in a dichotomous way (yes/no), strictly adhering to the criteria defined for the diagnostic classifications mentioned above.

### Covariates

We evaluated the following demographic and socioeconomic characteristics of the mother at the time of childbirth: age (≤ 19, 20–34 or ≥ 35 years); marital status (married/living with a partner or single/divorced/widowed); self-reported skin color (White or Black/mixed); parity (0, 1 or ≥ 2 previous pregnancies) and socioeconomic position (SEP).

SEP was determined on the basis of the wealth index and the maternal level of education. The wealth index can be used as a general indicator of material living standards. It takes into consideration durable consumer goods (e.g., televisions, cars, computers, and stoves) and household characteristics (e.g., number of toilets), through principal component analysis. For our analyses, the first component was selected and categorized in quintiles [25]. The maternal level of education was categorized as < 5, 5–8, or ≥ 9 years of formal schooling.

Mood symptoms during pregnancy were identified on the basis of an affirmative response to the question: “During pregnancy, did you feel depressed or have any nervous condition?” Smoking and alcohol use during pregnancy were self-reported and were evaluated retrospectively at birth. Regular smokers were defined as those women who smoked at least one cigarette daily in any trimester of pregnancy. Any amount of alcohol intake during any trimester of pregnancy was considered as alcohol consumption during pregnancy.

The following characteristics of the adolescent were evaluated at birth: gender; multiple pregnancy; 5-min Apgar score (< 7 or ≥ 7); low birthweight (LBW, < 2500 g); and preterm birth (< 37 weeks of gestational age).

Assessment of maternal depressive symptoms was made using the Edinburgh Postnatal Depression Scale (EPDS) [26]. The EPDS questionnaire comprises 10 questions that assess symptoms of depression in the last 7 days. The EPDS was administered to all of the mothers at each follow-up, except at the 3-month follow-up visit, when it was completed by a subsample of 965 mothers. Trajectories of maternal depressive symptoms in the first years of the adolescents´ life were estimated using a semiparametric group-based modeling, a form of finite mixture modeling proposed by Nagin & Odgers [27]. A censored normal model was adjusted to the data. Five trajectories of maternal depressive symptoms were estimated: low and moderate-low-symptoms, increasing, decreasing and high-chronic maternal depressive symptomatology [28].

### Statistical analysis

To describe the characteristics of the sample we used absolute and relative frequencies. To examine the association between psychiatric disorders and gender and SEP, psychiatric disorders were organized in the following groups: any psychiatric disorder; any anxiety disorder; any mood disorder; any ADHD/hyperactivity disorder; any conduct/oppositional disorder; any autism spectrum disorder; any tic disorder/Tourette syndrome and any eating disorder. The statistical tests were based on the Chi-square test.

Logistic regression was used to perform bivariate and multivariate analyses, the results of which were expressed as odds ratio (OR) and 95% CI. The adjusted analysis employed a conceptual hierarchical model to determine the risk factors for any psychiatric disorder. That model consisted of five levels: demographic and socioeconomic variables; variables related to maternal behavioral traits; mood symptoms during pregnancy; characteristics of the child at birth; and trajectories of maternal depressive symptoms between 3 months and 6 years after the birth of the child. The model considers the effect of each variable in relation of the outcome, controlling for confounding among variables of the same or higher level. Variables presenting *p* < 0.20 in the adjusted analysis were maintained in each hierarchical level.

Statistical analyses were performed using the program Stata, version 13 (StataCorp LP, College Station, TX).

### Ethical aspects

The study was approved by the Research Committee of the University of São Paulo School of Medicine, (Research Protocol no. 015/15), and by the Research Ethics Committee of the Federal University of Pelotas. Written informed consent was obtained from the mothers or legal guardians of the adolescents. At the 11-year follow-up, adolescents also signed an informed consent form. Cases of severe mental health problems, as identified by the psychologists, were evaluated and, when necessary, were referred to the psychiatric or psychological care facilities available in the city.

## Results

### Attrition analysis and characteristics of the sample

Of the 4231 participants constituting the original cohort, 98 died in the first 11 years of life and 3566 were interviewed at 11 years. Data about psychiatric disorders at 11 years were available on 3562 adolescents (84.2% of the original cohort). Mothers of participants who remained in the study were more wealthy, educated and less likely to be single, multiparous, smokers and to report mood symptoms during pregnancy (Table 1). The final sample also included a smaller proportion of subjects who had been low birthweight or preterm neonates, as well as fewer subjects who had a 5-min Apgar score < 7 at birth compared to those not included in the present analyses.

**Table 1:** Comparison of maternal and child characteristics between those included and not included in the present study, Pelotas 2004 birth cohort

Variables	Included (*n* = 3562)	Not included (*n* = 669)	*p* value*
Maternal characteristics			
Wealth index (quintiles)			0.013
1st (lowest) to 3st	2107 (59.1)	430 (64.3)	
4th to 5th (highest)	1456 (40.9)	238 (35.7)	
Maternal education (year)			0.007
<5	525 (14.9)	130 (19.7)	
5–8	1465 (41.6)	266 (40.2)	
≥9	1536 (43.6)	265 (40.1)	
Maternal age (year)			0.060
≤19	668 (18.8)	132 (19.7)	
20–34	2399 (67.4)	467 (69.8)	
≥35	493 (13.9)	70 (10.5)	
Marital status			0.001
Single/divorced/widowed	556 (15.6)	139 (20.8)	
Married/living with a partner	3006 (84.4)	530 (79.2)	
Maternal skin color			0.956
White	2602 (73.1)	488 (72.9)	
Black/mixed	960 (26.9)	181 (27.1)	
Parity			0.083
0	1406 (39.5)	260 (38.9)	
1	954 (26.8)	157 (23.5)	
≥2	1201 (33.7)	252 (37.7)	
Mood symptoms during pregnancy			< 0.001
No	2706 (76.0)	463 (69.2)	
Yes	854 (24.0)	206 (30.8)	
Smoking during pregnancy			0.008
No	2612 (73.3)	457 (68.3)	
Yes	950 (26.7)	212 (31.7)	
Alcohol during pregnancy			0.252
No	3449 (96.8)	642 (96.0)	
Yes	113 (3.2)	27 (4.0)	
Children's characteristics			
Gender			0.321
Male	1837 (51.6)	359 (53.7)	
Female	1725 (48.4)	310 (46.3)	
Multiple pregnancy			0.699
No	3490 (98.0)	657 (98.2)	
Yes	72 (2.0)	12 (1.8)	
5-min Apgar score			< 0.001
≥7	3488 (98.4)	624 (94.5)	
<7	56 (1.6)	36 (5.5)	
Low birthweight			< 0.001
No	3241 (91.0)	563 (84.4)	
Yes	320 (9.0)	104 (15.6)	
Preterm birth			0.006
No	3063 (86.1)	541 (82.0)	
Yes	494 (13.9)	119 (18.0)	

* *χ*^2^ test

Amongst included participants, 44% of the mothers had studied 9 or more years and nearly 70% of them were between 20- and 34-years-old when the adolescent was born. For 40% of the mothers, the cohort participant was the first child, and 16% of them were not living with a partner at the time of delivery. Almost 27% of the mothers smoked during pregnancy, 3% consumed any alcohol beverage and one-fourth of them reported mood symptoms during pregnancy. At birth, approximately 52% of the subjects were male, 2% were twins, 10% were LBW neonates, 14% premature and less than 2% presented a 5-min Apgar score < 7.

### Prevalence of psychiatric disorders

We found that 13.2 and 11.3% of the adolescents, respectively, met at least one DSM-5 or ICD-10 diagnostic criterion for a psychiatric disorder (Table 2).

**Table 2:** Prevalence of psychiatric disorders among 11-year-olds, according to the DSM-5 and ICD-10 criteria, Pelotas 2004 birth cohort (*n* = 3562)

Psychiatric disorder	DSM-5		ICD-10	
	*n*	% (95% CI)	*n*	% (95% CI)
Any psychiatric disorder	471	13.2 (12.1–14.4)	401	11.3 (10.2–12.3)
Any anxiety disorder	154	4.3 (3.7–5.0)	134	3.8 (3.2–4.4)
Separation anxiety	41	1.2 (0.8–1.6)	22	0.6 (0.4–0.9)
Specific phobia	63	1.8 (1.4–2.3)	62	1.7 (1.3–2.2)
Social phobia	14	0.4 (0.2–0.7)	13	0.4 (0.2–0.6)
Panic disorder	5	0.1 (0.05–0.3)	5	0.1 (0.05–0.3)
Agoraphobia	7	0.2 (0.1–0.4)	7	0.2 (0.08–0.4)
Post-traumatic stress disorder	8	0.2 (0.1–0.4)	7	0.2 (0.08–0.4)
Obsessive–compulsive disorder	8	0.2 (0.1–0.4)	5	0.1 (0.05–0.3)
Body dysmorphic disorder	8	0.2 (0.1–0.4)	–	–
Generalized anxiety disorder	15	0.4 (0.2–0.7)	16	0.5 (0.3–0.7)
Other anxiety disorder	13	0.4 (0.2–0.6)	13	0.4 (0.2–0.6)
Any mood disorder	115	3.2 (2.7–3.9)	42	1.2 (0.9–1.6)
Disruptive mood dysregulation disorder	86	2.4 (1.9–3.0)	–	–
Major depressive disorder	20	0.6 (0.3–0.9)	–	–
Other depressive disorder	10	0.3 (0.1–0.5)	10	0.3 (0.1–0.5)
Mild depressive episode	–	–	6	0.2 (0.1–0.4)
Moderate depressive episode	–	–	13	0.4 (0.2–0.6)
Severe depressive episode	–	–	4	0.1 (0.03–0.3)
Mania/bipolar disorder	12	0.3 (0.2–0.6)	12	0.3 (0.2–0.6)
Any ADHD/hyperactivity disorder	144	4.0 (3.4–4.7)	120	3.4 (2.8–4.0)
Combined-type ADHD	74	2.1 (1.6–2.6)	–	–
Inattentive-type ADHD	35	1.0 (0.7–1.4)	–	–
Hyperactive/impulsive-type ADHD	16	0.5 (0.3–0.7)	–	–
Hyperkinesia	–	–	73	2.1 (1.6–2.6)
Other hyperactive/hyperkinetic disorder	19	0.5 (0.3–0.8)	47	1.3 (1.0–1.8)
Any conduct/oppositional disorder	98	2.8 (2.2–3.3)	118	3.3 (2.7–4.0)
Oppositional defiant disorder	68	1.9 (1.5–2.4)	109	3.1 (2.5–3.7)
Conduct disorder	35	1.0 (0.7–1.4)		
Conduct disorder confined to family	–	–	2	0.1 (0.01–0.2)
Unsocialized conduct disorder	–	–	6	0.2 (0.01–0.4)
Socialized conduct disorder	–	–	26	0.7 (0.5–1.1)
Other conduct/oppositional disorder	14	0.4 (0.2–0.7)	11	0.3 (0.2–0.6)
Any autism spectrum disorder	15	0.4 (0.2–0.7)	14	0.4 (0.2–0.7)
Autism spectrum disorder	12	0.3 (0.2–0.6)	11	0.3 (0.2–0.6)
Other autism spectrum disorder	3	0.1 (0.02–0.2)	3	0.08 (0.02–0.2)
Any tic disorder	55	1.5 (1.2–2.0)	40	1.1 (0.8–1.5)
Tourette syndrome	12	0.3 (0.2–0.6)	9	0.3 (0.1–0.5)
Chronic tic disorder	41	1.2 (0.8–1.6)	27	0.8 (0.5–1.1)
Other tic disorder	2	0.06 (0.01–0.2)	4	0.1 (0.03–0.3)
Any eating disorder	18	0.5 (0.3–0.8)	18	0.5 (0.3–0.8)
Anorexia nervosa	5	0.1 (0.05–0.3)	5	0.1 (0.05–0.3)
Eating disorder not otherwise specified	1	0.03 (0.00–0.2)	13	0.4 (0.2–0.6)
Binge-eating disorder	12	0.3 (0.2–0.6)	–	–

*ADHD* attention deficit/hyperactivity disorder

The most common psychiatric diagnoses were anxiety disorders, which were identified in approximately 4% of the adolescents. The most common anxiety disorders were specific phobia and separation anxiety. The prevalence of depressive disorders was 0.8% according to the DSM-IV criteria, 3.0% according to the DSM-5 criteria (which include DMDD), and 1.2% according to the ICD-10 criteria. The prevalence of DMDD and body dysmorphic disorder, categories present only in the DSM-5, was 2.4 and 0.2%, respectively. The overall prevalence of mood disorders was 3.2% when the DSM-5 criteria were applied and 1.2% when the ICD-10 criteria were applied. According to the DSM-5 criteria, the prevalence of ADHD or other hyperactivity disorder was 4.0%, the combined type of ADHD being predominant (occurring in 2.1%). Using the ICD-10 diagnostic classification, we found that the overall prevalence of ADHD or other hyperkinetic disorder was 3.4%, that of hyperkinesia being 2.1% and that of ADHD not otherwise specified being 1.3%. The prevalence of conduct disorder and ODD was 2.8 and 3.3% according to the DSM-5 and ICD-10, respectively. Autistic spectrum disorders and eating disorders were rarely found among the adolescents.

### Risk factors for psychiatric disorders

The proportion of adolescents with at least one psychiatric disorder was greater among the boys than among the girls (Table 3). The prevalence of any ADHD/ hyperactivity disorder, any conduct/oppositional disorder, and any tic disorder/Tourette syndrome was nearly twice as high among male subjects as among female subjects. Although low in both genders, the prevalence of eating disorders was higher among the girls than among the boys.

**Table 3:** Prevalence of psychiatric disorders among 11-year-olds, by DSM-5 and ICD-10 categories, according to the adolescent sex, Pelotas 2004 birth cohort

Category	DSM-5			CID-10		
	Boys	Girls	*p* value*	Boys	Girls	*p* value*
	*n* (%)	*n* (%)		*n* (%)	*n* (%)	
Any psychiatric disorder	287 (15.6)	184 (10.7)	< 0.001	247 (13.5)	154 (8.9)	< 0.001
Any anxiety disorder	79 (4.3)	75 (4.3)	0.959	67 (3.7)	67 (3.9)	0.723
Any mood disorder	69 (3.8)	46 (2.7)	0.064	24 (1.3)	18 (1.0)	0.462
Any ADHD/hyperactivity disorder	106 (5.8)	38 (2.2)	< 0.001	93 (5.1)	27 (1.6)	< 0.001
Any conduct/oppositional disorder	69 (3.8)	29 (1.7)	< 0.001	83 (4.5)	35 (2.0)	< 0.001
Any autism spectrum disorder	13 (0.7)	2 (0.1)	0.006	12 (0.7)	2 (0.1)	0.010
Any tic disorder/Tourette syndrome	43 (2.3)	12 (0.7)	< 0.001	31 (1.7)	9 (0.5)	0.001
Any eating disorder	5 (0.3)	13 (0.8)	0.043	5 (0.3)	13 (0.8)	0.043

*ADHD* attention deficit/hyperactivity disorder

*Chi-square test

The prevalence of any psychiatric disorder, any depressive disorder, any ADHD/ hyperactivity disorder, and any conduct/oppositional disorder was lower among adolescents belonging to wealthier families than among those belonging to poorer families (Table 4). The prevalence of any psychiatric disorder, any depressive disorder, any ADHD/hyperactivity disorder, and any conduct/oppositional disorder was lower among adolescents whose mothers had ≥ 9 years of schooling than among those whose mothers had < 9 years of schooling.

**Table 4:** Prevalence of psychiatric disorders among 11-year-olds, by DSM-5 category according to the socioeconomic position (Wealth Index and maternal level of education), Pelotas 2004 birth cohort

Category	Wealth index (quintile), *n* (%)						Maternal years of schooling, *n* (%)			
	1	2	3	4	5	p*	< 5	5–8	≥ 9	p*
Any psychiatric disorder	112 (17.4)	103 (13.8)	88 (12.3)	100 (13.4)	67 (9.5)	< 0.001	98 (18.7)	194 (13.2)	173 (11.3)	< 0.001
Any anxiety disorder	33 (5.1)	29 (3.9)	32 (4.5)	33 (4.4)	27 (3.8)	0.418	26 (5.0)	58 (4.0)	69 (4.5)	0.916
Any mood disorder	27 (4.2)	34 (4.6)	21 (2.9)	21 (2.8)	12 (1.7)	0.001	36 (6.9)	53 (3.6)	25 (1.6)	< 0.001
Any ADHD/hyperactivity disorder	35 (5.4)	41 (5.5)	24 (3.3)	28 (3.7)	16 (2.3)	0.001	35 (6.7)	58 (4.0)	50 (3.3)	0.003
Any conduct/oppositional disorder	27 (4.2)	29 (3.9)	18 (2.5)	17 (2.3)	7 (1.0)	< 0.001	24 (4.6)	45 (3.1)	27 (1.8)	< 0.001
Any autism spectrum disorder	3 (0.5)	3 (0.4)	2 (0.3)	6 (0.8)	1 (0.1)	0.773	2 (0.4)	8 (0.6)	5 (0.3)	0.634
Any tic disorder/Tourette syndrome	12 (1.9)	9 (1.2)	13 (1.8)	8 (1.1)	13 (1.8)	0.920	7 (1.3)	24 (1.6)	23 (1.5)	0.922
Any eating disorder	3 (0.5)	2 (0.3)	6 (0.8)	5 (0.7)	2 (0.3)	0.954	3 (0.6)	7 (0.5)	8 (0.5)	0.956

*ADHD* attention deficit/hyperactivity disorder

*Chi square test

In the adjusted analysis, the odds of developing any psychiatric disorder by age 11 was higher among adolescents of less educated women, smokers and those with depressive symptoms during pregnancy (Table 5). Adolescents of mothers in the “high-chronic” depressive trajectory group were more than four times as likely to have any psychiatric disorder compared to adolescents of women in the “low” group. Male gender, 5-min Apgar score < 7 at birth and preterm birth were associated with higher risk of any psychiatric disorder at age 11.

**Table 5:** Crude and adjusted analyses of the association between maternal and child's characteristics and any psychiatric disorder at age 11 according to DSM-5, Pelotas 2004 birth cohort

Variable	Crude analysis		Adjusted analysis	
	OR (95% CI)	*p* value	OR (95% CI)	*p* value
Level 1—demographic and socioeconomic variables				
Wealth index (quintiles)		< 0.001		0.152
1st (lowest)	2.0 (1.5; 2.8)		1.6 (1.1; 2.3)	
2nd	1.5 (1.1; 2.1)		1.3 (0.9; 1.9)	
3rd	1.3 (1.0; 1.9)		1.2 (0.8; 1.7)	
4th	1.5 (1.1; 2.0)		1.4 (1.0; 2.0)	
5th (highest)	1.0 (Reference)		1.0 (Reference)	
Maternal education (year)		< 0.001		0.042
<5	1.8 (1.4; 2.4)		1.4 (1.0; 1.9)	
5–8	1.2 (1.0; 1.5)		1.0 (0.8; 1.3)	
≥9	1.0 (Reference)		1.0 (Reference)	
Maternal age (year)		0.090		0.128
≤19	1.2 (0.9; 1.5)		1.2 (0.9; 1.6)	
20–34	1.0 (Reference)		1.0 (Reference)	
≥35	0.8 (0.6; 1.1)		0.8 (0.6; 1.1)	
Marital status		0.051		0.121
Married/living with a partner	1.0 (Reference)		1.0 (Reference)	
Single/divorced/widowed	1.3 (1.0; 1.7)		1.2 (0.9; 1.6)	
Maternal skin color		0.072		0.507
White	1.0 (Reference)		1.0 (Reference)	
Black/mixed	1.2 (0.9; 1.5)		1.1 (0.9; 1.3)	
Parity		0.020		0.064
0	1.2 (0.9; 1.5)		1.1 (0.8; 1.4)	
1	1.0 (Reference)		1.0 (Reference)	
≥2	1.4 (1.1; 1.8)		1.4 (1.0; 1.8)	
Level 2—variables related to maternal behavioral traits				
Smoking during pregnancy		< 0.001		0.021
No	1.0 (Reference)		1.0 (Reference)	
Yes	1.5 (1.2; 1.9)		1.3 (1.0; 1.6)	
Alcohol during pregnancy		0.058		0.139
No	1.0 (Reference)		1.0 (Reference)	
Yes	1.6 (1.0; 2.6)		1.4 (0.9; 2.4)	
Level 3—Moods symptoms during pregnancy				
Mood symptoms during pregnancy		< 0.001		< 0.001
No	1.0 (Reference)		1.0 (Reference)	
Yes	1.5 (1.2; 1.9)		1.4 (1.1; 1.7)	
Level 4—Child's characteristics at birth				
Gender		< 0.001		< 0.001
Male	1.0 (Reference)		1.0 (Reference)	
Female	0.6 (0.5; 0.8)		0.6 (0.5; 0.8)	
Multiple pregnancy		0.859		0.606
No	1.0 (Reference)		1.0 (Reference)	
Yes	0.9 (0.5; 1.9)		0.8 (0.4; 1.7)	
5-min Apgar score		0.010		0.028
≥7	1.0 (Reference)		1.0 (Reference)	
<7	2.2 (1.2; 4.1)		2.1 (1.1; 3.9)	
Low birthweight		0.097		0.620
No	1.0 (Reference)		1.0 (Reference)	
Yes	1.3 (1.0; 1.8)		1.1 (0.8; 1.6)	
Preterm birth		0.009		0.044
No	1.0 (Reference)		1.0 (Reference)	
Yes	1.4 (1.1; 1.8)		1.3 (1.0; 1.7)	
Level 5—Trajectories of maternal depressive symptoms in the first years of the adolescents' life				
Trajectories of maternal depressive symptoms		< 0.001		< 0.001
Low	1.0 (Reference)		1.0 (Reference)	
Moderate—low	1.9 (1.4; 2.5)		1.7 (1.3; 2.4)	
Increasing	3.9 (2.7; 5.6)		3.4 (2.3; 5.1)	
Decreasing	3.9 (2.7; 5.6)		3.5 (2.4; 5.2)	
High-chronic	5.3 (3.5; 8.1)		4.7 (3.0; 7.5)	

### Psychiatric comorbidity

Among adolescents with psychiatric disorders (*n* = 471), two psychiatric disorders were observed in 73 (15.5%), three were observed in 24 (5.1%) and four psychiatric disorders were observed in 10 (2.1%).

The various diagnostic combinations are detailed in the Electronic Supplementary Material (Table 1). Among the 73 adolescents diagnosed with two psychiatric disorders, the most common combination was any mood disorder with any ADHD/hyperactivity disorder, which was seen in 18 (24.6%), followed by any anxiety disorder with any mood disorder, seen in 16 (21.9%), any ADHD/hyperactivity disorder with any conduct/oppositional disorder, seen in 13 (17.8%), and any anxiety disorder with any ADHD/ hyperactivity disorder, seen in 10 (13.7%).

## Discussion

The prevalence of a diagnosis of any psychiatric disorder at age 11 was 13.2 and 11.3%, respectively, when the DSM-5 and ICD-10 criteria were applied. The prevalence of any psychiatric disorder was higher among the male adolescents, mainly because ADHD/hyperactivity disorders and conduct/oppositional disorders were more common among the boys. The prevalence of any mood disorder, any ADHD/ hyperactivity disorder, and any psychiatric disorder was higher among adolescents belonging to families with a lower SEP than among those belonging to families with a higher SEP. Low maternal education, smoking during pregnancy, the presence of moods symptoms during pregnancy or maternal chronic and severe depressive symptoms in the first years of the adolescent´s life, male gender, 5-min Apgar score < 7 at birth and preterm birth were associated with higher odds of any psychiatric disorder at age 11.

The prevalence of psychiatric disorders among the adolescents in our sample is comparable to that reported in a recent systematic review and meta-analysis that included studies conducted in 27 countries and employed various methods of assessing psychiatric disorders [8]. Other studies from low- and middle-income countries, in which the DAWBA was applied, reported prevalence rates similar to those of our study. In a two-stage prevalence study of randomized samples of children between 5 and 10 years of age (*n* = 922) from three contrasting areas of Bangladesh [24], the overall prevalence of any psychiatric disorder was 15.2% (15.4% in rural areas, 10.0% in urban areas with a better SEP, and 19.5% in urban areas with a worse SEP). In a sample of 448 subjects between 7 and 14 years of age in Russia [29], the observed prevalence of psychiatric disorders was 15.3%, 70% higher than that previously observed in a comparable study of children and adolescents in Great Britain [30]. In a study involving subjects between 7 and 14 years of age in the southeastern region of Brazil [23], the reported prevalence of psychiatric disorders was 12.7%.

The rates of psychiatric disorders are generally higher in studies using screening instruments than in those using diagnostic instruments. A study conducted in the city of Taubaté, in the state of São Paulo, Brazil, evaluated 454 school children (7–11 years of age) at public and private schools [31]. Using the Strengths and Difficulties Questionnaire, the authors found that 35.2% of the subjects were considered positive for mental health problems, which reached clinical relevance in 22.7% and borderline relevance in 12.5%. The *Estudo de Riscos Cardiovasculares em Adolescentes* (ERICA, Study of Cardiovascular Risk in Adolescents), a cross-sectional school-based study conducted in Brazilian municipalities with more than 100,000 inhabitants, evaluated 74,589 adolescents [32]. Using the 12-item General Health Questionnaire, the authors found that, among the subjects between 12 and 14 years of age, the prevalence of common psychiatric disorders was 26.7%.

In the present study, the evaluation of the prevalence and symptomatology of psychiatric disorders was performed according to the DSM-5 criteria. Therefore, in addition to the specific differences between the DSM-IV and DSM-5, we included three nosological categories belonging exclusively to the DSM-5: DMDD; body dysmorphic disorder; and binge-eating disorder. DMDD, which is included in the DSM-5 depressive disorders section, was the main factor accounting for the difference of mood disorders prevalence between the DSM-5 and ICD-10 criteria. It should be noted that, according to the DSM-5, the symptoms of ODD often occur in children with DMDD, the main difference being that angry outbursts and the occurrence of the symptoms in more than one sphere (family, school, and social) are more common in the latter. Therefore, this new diagnostic category (DMDD) also explains the difference between prevalence rates for conduct/oppositional disorders when ICD-10 and DSM-5 are used.

In our study, the most common psychiatric disorders were anxiety disorders. A systematic review of 11 studies, using the DSM-III, Revised or DSM-IV diagnostic criteria, showed that the reported prevalence of any anxiety disorder in children under 12 years of age ranged from 2.6 to 41.2% [33]. The authors found that the most common anxiety diagnosis in that age group was separation anxiety disorder. Despite the notable variation in prevalence estimates, which is likely due to differences in methodology and the instruments used, the lifetime prevalence of “any anxiety disorder” in studies involving children or adolescents is between 15 and 20% [34].

In the present study, the prevalence of any ADHD/ hyperactivity disorder was similar to estimates reported for children and adolescents around the world (5.3% in individuals under 18 years of age) [19]. A recent cross-sectional study that assessed 1676 6-to-16 year-olds from four regions of Brazil using the Schedule for Affective Disorders and Schizophrenia for School-Age Children/Present-and-Lifetime-Version (K-SADS-PL) applied to mothers/main caregivers, reported a prevalence of any ADHD/hyperactivity disorder of 4.5% (CI 95% 3.5–5.6) [35].

Epidemiological and clinical studies have found gender-related differences in many types of psychopathology. Early-onset disorders (those that begin in childhood) are typically more prevalent in boys, whereas those that begin in adolescence are more prevalent in girls [36]. Such differences vary by age group. For example, most studies involving children report that the prevalence of conduct disorders, with aggressive and antisocial behaviors, is higher among boys than among girls. During adolescence, the prevalence of depression and eating disorders is higher among girls, who are also more likely to engage in suicidal ideation. Adolescent boys, however, have more problems with anger, more often engage in high-risk behaviors, and are more likely to commit suicide [37]. Contrary to what was observed in the ERICA study, in which the prevalence of common psychiatric disorders in subjects 12–14 years of age was higher in girls than in boys, in our study the prevalence of any psychiatric disorder was higher in the boys than in the girls [32]. Because our study involved subjects aged 11 years, the frequency of disorders by gender found in the current study may reflect the prevalence of disorders observed in childhood, which is different from the prevalence of disorders by gender observed in older adolescents.

The SEP is used in epidemiological studies as a measure of socioeconomic factors that influence the place an individual occupies within the structure of society [38]. Although there are several indicators of the SEP, schooling, income, and the wealth index are the most widely used. Poverty is associated with multiple environmental risk factors for psychopathology, such as parental unemployment, maternal anxiety/depression, child/adolescent maltreatment and trauma exposure and fewer resources to access good quality health care. A systematic review that evaluated the effects of the SEP on the occurrence of psychiatric disorders in children and adolescents reported that the prevalence of psychiatric disorders was higher among children and adolescents belonging to families with a low income and whose parents had a low level of education than among those whose parents had higher incomes or higher levels of education [39]. In our study, after adjustment for potential confounders, only poor maternal schooling was associated with higher risk of any psychiatric disorder at age 11.

In accordance with our results, many studies reported the association between fetal exposure to maternal smoking in pregnancy and several adverse offspring mental health outcomes [40, 41]. Recently, Talati et al. showed that birthweight did not mediate the association between the exposure and externalizing psychopathology, indicating that the mechanisms through which maternal smoking increases the risk of offspring psychopathology were not operating through lower birthweight [42].

Consistent with previous reports, we found that maternal depression, especially when it is chronic and severe, was a strong predictor of offspring psychiatric disorders [43, 44]. Even though the exposure to maternal depression appears to be a significant independent risk factor for offspring mental disorders, Barker et al. reported that multiple risk factors exposures strongly affect child psychopathology, increasing the risk over and above the influence of maternal depression [45].

In our study, both preterm birth and 5-min Apgar score < 7 were associated with psychiatric disorders at age 11. The Apgar score is widely used to report the status of the newborn infant immediately after birth. Even though numerous factors can influence the Apgar score, 5-min low Apgar scores have been associated with an increased risk of a wide range of neurological and psychiatric disorders [46, 47]. Preterm birth has also been identified as a risk factor for several psychiatric disorders in childhood, such as emotional problems, ADHD and autism spectrum, increasing the risk significantly as gestational age decreases [48, 49].

In the present study, 22.7% of the adolescents with psychiatric disorders had one or more psychiatric comorbidities. Anxiety disorders accompany most psychiatric disorders, including mood disorders, disruptive behaviors, eating disorders, and substance use disorders. The combination of anxiety disorders and mood disorders is so common that many authors have postulated that anxiety disorders are part of the developmental sequence in which anxiety is expressed early in life, followed by depression in adulthood [34]. Community studies in young people have shown a high degree of the co-occurrence of conduct disorders and ADHD, which is associated with a worse prognosis in behavioral disorders, including substance use disorders [50]. Disruptive behavior disorders are also frequently accompanied by mood and anxiety disorders, although parents and teachers often report fewer problems related to anxiety and mood than to externalizing disorders, which are more easily perceived and more difficult to manage. The presence of psychiatric comorbidity interferes with the evolution of the psychiatric disorder, making its course more chronic, with a worse prognosis and a worse response to treatment [18]. The prevalence of comorbidities tends to be higher in studies conducted at mental health facilities and such comorbidities tend to occur more frequently in the later stages of development. A recent study by Bordin et al. showed that comorbidity increased the likelihood of maternal recognition of emotional/behavioral problems in children and adolescents, an essential first step in the searching for treatment or support for their children [51].

### Strengths and limitations of the study

Among the advantages of the study is that it was a population-based study, with face-to-face interviews, involving a large sample of adolescents. In addition, we used an internationally recognized instrument, designed to generate diagnoses of psychiatric disorders, that has been validated for use in Brazil and was applied by trained psychologists, thus ensuring the quality of the data. Another important point is that we employed the criteria of the recently published DSM-5, which follows internationally accepted diagnostic criteria. Furthermore, the proportion of non-respondents was low.

One limitation of our study is that it was based only on information obtained from the mothers or legal guardians, because the DAWBA was not administered to the adolescents themselves or to their teachers. Reports from teachers and self-reports from adolescents could reveal other symptoms not recognized by the mothers, contributing to a more accurate diagnosis of the psychiatric disorders.

## Conclusions

Our findings underscore the relevance of psychiatric disorders in early adolescence and provide evidence for the development of strategies for the prevention of psychiatric disorders, as well as for the promotion and recovery of mental health, during childhood and adolescence. We also observed a higher frequency of psychiatric disorders in boys and in the adolescents of mothers with chronic depression and with a less favorable SEP. Our results might contribute to reducing the knowledge gap related to the lack of population-based studies on psychiatric disorders in adolescents in low- and middle-income countries, as well as facilitating the planning of specific services to address psychiatric disorders in this age group, minimizing the short- and long-term impact of such disorders along the life-course.

## Electronic supplementary material

Below is the link to the electronic supplementary material.

Supplementary material 1 (DOCX 13 KB)
