# Supplementary material for: Prevalence and risk factors of psychiatric disorders in early adolescence: 2004 Pelotas (Brazil) birth cohort
Source: Soc Psychiatry Psychiatr Epidemiol. 2018 Apr 13;53(7):685–97. doi: 10.1007/s00127-018-1516-z (PMC6003978; doi:10.1007/s00127-018-1516-z)
Supplement: Supplementary file 1 — Supplementary material 1 (DOCX 13 KB) [file 127_2018_1516_MOESM1_ESM.docx]

**Social Psychiatry and Psychiatric Epidemiology**

**Electronic supplementary material**

**Prevalence and Risk Factors of Psychiatric Disorders in Early Adolescence: 2004 Pelotas (Brazil) Birth Cohort**

Carolina La Maison, MSc,^1^  Tiago N. Munhoz, PhD,^2,3^ Iná S. Santos, MD, PhD,^3^ Luciana Anselmi, PhD,^3^ Fernando C. Barros, MD, PhD,^4^ Alicia Matijasevich, MD, PhD,^1,3^

^1^ Department of Preventive Medicine, School of Medicine, University of São Paulo, São Paulo, Brazil

^2^ Department of Psychology, Federal University of Pelotas, Pelotas, Brazil

^3^ Postgraduate Program in Epidemiology, Federal University of Pelotas, Pelotas, Brazil

4 Postgraduate Program in Health and Behavior, Catholic University of Pelotas, Pelotas, Brazil

**Corresponding Author**: Tiago N Munhoz. Centro de Pesquisas Epidemiológicas - Universidade Federal de Pelotas, Rua Marechal Deodoro, CEP: 96020-220- Caixa Postal 464, 1160, Pelotas, RS, Brasil. Tel./fax: +55 5332841300.

E-mail address: tiago.munhoz@ufpel.edu.br (TN Munhoz).

**Key Words**: Mental Disorders; Neurodevelopmental Disorders; Prospective Studies; Adolescent.

**TABLE 1. Psychiatric Comorbidities, according to the DSM-5 categories, in 11-year-olds, Pelotas 2004 Birth Cohort (n=471)**

| **2 comorbidities (n=73)** | **n** |
| --- | --- |
| Any mood disorder + any ADHD/ hyperactivity disorder | 18 |
| Any anxiety disorder + any mood disorder | 16 |
| Any ADHD/ hyperactivity disorder + any conduct/oppositional disorder | 13 |
| Any anxiety disorder + any ADHD/ hyperactivity disorder | 10 |
| Any mood disorder + any conduct/oppositional disorder | 4 |
| Any anxiety disorder + any conduct/oppositional disorder | 3 |
| Any anxiety disorder + any tic disorder | 3 |
| Any conduct/oppositional disorder + any eating disorder | 2 |
| Any ADHD/ hyperactivity disorder + any autism spectrum disorder | 1 |
| Any ADHD/ hyperactivity disorder + any tic disorder | 1 |
| Any conduct/oppositional disorder + any autism spectrum disorder | 1 |
| Any conduct/oppositional disorder + any tic disorder | 1 |
| **3 comorbidities (n=24)** | **n** |
| Any mood disorder + any ADHD/ hyperactivity disorder + any conduct/oppositional disorder | 10 |
| Any anxiety disorder + any mood disorder + any ADHD/ hyperactivity disorder | 4 |
| Any anxiety disorder + any ADHD/ hyperactivity disorder + any conduct/oppositional disorder | 4 |
| Any ADHD/ hyperactivity disorder + any conduct/oppositional disorder + any autism spectrum disorder | 2 |
| Any mood disorder + any ADHD/ hyperactivity disorder + any autism spectrum disorder | 2 |
| Any anxiety disorder + any mood disorder + any conduct/oppositional disorder | 1 |
| Any anxiety disorder + any mood disorder + any eating disorder | 1 |
| **4 comorbidities (n=10)** | **n** |
| Any anxiety disorder + any mood disorder + any ADHD/ hyperactivity disorder + any conduct/oppositional disorder | 4 |
| Any mood disorder + any ADHD/ hyperactivity disorder + any conduct/oppositional disorder + any tic disorder | 3 |
| Any anxiety disorder + any mood disorder + any ADHD/ hyperactivity disorder + any tic disorder | 2 |
| Any anxiety disorder + any mood disorder + any ADHD/ hyperactivity disorder + any autism spectrum disorder | 1 |

ADHD=attention deficit/hyperactivity disorder.
